# The effect of decreasing human activity from COVID‐19 on the foraging of fallen fruit by omnivores

**DOI:** 10.1002/ece3.9657

**Published:** 2022-12-25

**Authors:** Shigeru Osugi, Seungyun Baek, Tomoko Naganuma, Kahoko Tochigi, Maximilian L. Allen, Shinsuke Koike

**Affiliations:** ^1^ United Graduate School of Agricultural Science Tokyo University of Agriculture and Technology Fuchu, Tokyo Japan; ^2^ Institute of Global Innovation Research Tokyo University of Agriculture and Technology Fuchu, Tokyo Japan; ^3^ Illinois Natural History Survey, University of Illinois Champaign Illinois USA

**Keywords:** activity pattern, anthropause, human impact, lockdown, omnivore

## Abstract

In 2020, a lockdown was implemented in many cities around the world to contain the COVID‐19 pandemic, resulting in a significant cessation of human activity which have had a variety of impacts on wildlife. But in many cases, due to limited pre‐lockdown information, and there are limited studies of how lockdowns have specifically affected behaviors. Foraging behavior is inherently linked to fitness and survival, is particularly affected by changes in temporal activity, and the influence of human disturbance on foraging behavior can be assessed quantitatively based on foraging duration and quantity. The purpose of this study was to determine whether and how the fruit‐foraging behaviors of two omnivores, the Japanese badger (*Meles anakuma*) and the raccoon dog (*Nyctereutes procyonoides*), were influenced by the decrease of human activity associated with lockdowns. Specifically, by comparing to a previous study in 2019–2020, we attempted to determine (1) whether foraging behavior increases during the daytime? (2) whether the duration of foraging per visit increases? and (3) what factors animals select for in fruiting trees? The results of the initial investigation showed that the foraging behavior of both species in 2019 was almost exclusively restricted to the nighttime. But as opportunities for foraging behavior without human interference increased in 2020 due to the lockdown, both species (but especially raccoon dogs) showed substantial changes in their activity patterns to be more diurnal. The duration of foraging per visit also increased in 2020 for both species, and the selection during foraging for both species shifted from selecting trees that provided greater cover in 2019 to trees with high fruit production in 2020. Our results show how human activity directly affects the foraging behavior of wildlife in an urban landscape.

## Graphical abstract

We were able to show in detail the specific behavioral changes in the foraging behavior of urban mammals, especially raccoon dogs, due to changes in human activity after the voluntary lockdown due to the COVID‐19 pandemic.

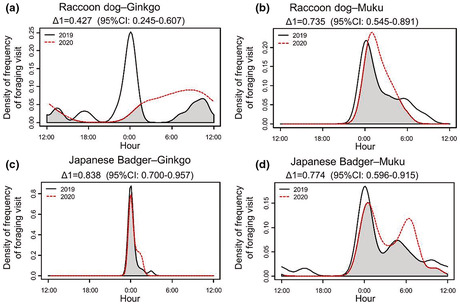

## 1 INTRODUCTION

Human activities are well known to have substantial effects on the activity and behavior of wildlife (Ditchkoff et al., 2006; Sol et al., 2013; Suraci et al., 2021). For example, since humans tend to be especially active in the daytime, most mammals shift their activity to be more nocturnal in response to human disturbance across all continents and habitats (Gaynor et al., 2018). Foraging behavior (often assessed quantitatively based on the duration and quantity of foraging) is inherently linked to fitness and survival, and is particularly affected by human activity and disturbance. Previous studies have reported that the presence of humans around foraging sites shortens the duration of foraging or alters the foraging sites for some animals (tiger, *Panthera tigris*; Kerley et al., 2002, puma, *Puma concolor*; Smith et al., 2015). In addition, frugivorous animals generally select to forage from trees with high fruit production; but in urban areas, some mammals choose trees with greater shelter rather than higher fruit production (Osugi et al., 2022) indicating that being able to avoid humans can outweigh foraging effectiveness.

In 2020, a lockdown was implemented in many cities around the world to contain the COVID‐19 pandemic, resulting in a significant cessation of human activity (“anthropause,” Rutz et al., 2020). Lockdowns around the world have had a variety of impacts on wildlife. For example, a reduction in the number of wildlife–vehicle collisions due to the reduced amount of traffic has been reported in many places (Bíl et al., 2021; Pokorny et al., 2022). It has also been reported that there has been an increase in the number and diversity of bird species, as well as changed behaviors, due to the decline in human outdoor activities (Gilby et al., 2021; Gordo et al., 2021). Many studies have reported on this unprecedented opportunity to understand how a massive change in human activity due to COVID‐19 affects wildlife behavior (Corlett et al., 2020; Manenti et al., 2020; Vardi et al., 2021). However, due to limited pre‐lockdown information, in many cases the discussion is based on suboptimal data not initially collected for this reason (information from surveillance cameras and statistical information). As a result, these studies can only indirectly report and speculate on the effects of lockdown on specific animal behaviors (Montgomery et al., 2021), and there are limited reports on how lockdowns have specifically affected behaviors such as foraging (Manenti et al., 2020).

We therefore used a university campus as our study area to investigate the impact of reduction in human activity from the COVID‐19 pandemic on foraging behavior by omnivores. In Japan, human activity was greatly reduced in 2020 in order to contain the COVID‐19 pandemic. In particular, there were repeated periods of greatly reduced human activity, accompanied by a sharp increase in the number of infected people every few months, with periods of attempted gradual recovery of economic activity in between. Meanwhile, universities implemented room entry restrictions for most of 2020, beginning with the implementation of online lectures, and students were absent from campus for the majority of the period. Therefore, the university is an ideal study site to examine the effects of the COVID‐19‐induced decline in human activity, as it became one of the places where human activity declined most rapidly and substantially in Japan (e.g., Arimura et al., 2020).

The purpose of this study was to determine whether the frugivory behavior of two species of omnivores, the Japanese badger (*Meles anakuma*) and the raccoon dog (*Nyctereutes procyonoides*), were affected by a sudden decrease in human activity. Although these two species are carnivores, they are omnivorous throughout the year and fruit is their staple food, especially in the fall and winter when insects are less abundant and the proportion of fruit in their diet is extremely high (e.g., Akihito et al., 2016; Kaneko et al., 2006). In addition, since these two species are not good tree climbers, their foraging for fruit is mainly for fallen fruit under fruiting trees. The objective of our study was to determine the specific and quantitative effects of changes in human activity on foraging behavior. In this context, the foraging behavior of fallen fruit is the most suitable for clarifying the effects of changing human activities alone, because it occurs only under fruiting trees, is easy to accurately observe, and the amount of fruit present (fruit production) as an environmental condition can be easily determined. Specifically, this study examined the foraging behavior on fallen fleshy fruits of these two species in a university campus in Tokyo, Japan where strict entry restrictions were in place in 2020, compared to the foraging behavior in 2019 before restrictions (Osugi et al., 2022).

Raccoon dogs and Japanese badgers have been living on this campus for many years. In the previous study that investigated the foraging behavior for fallen fruit in 2019, both Japanese badgers and raccoon dogs foraged mainly at night in urban forests compared to mountainous forest where there is almost no human activity. Furthermore, compared to mountainous forests, urban forests had significantly shorter duration of foraging per visit to fruiting trees. They also selected trees when foraging for fallen fruits that were in sheltered places where the forest floor was covered with vegetation, and fruit production did not affect tree choice. This indicates that the mammals in urban forests with high human activity normally prioritize avoiding humans over foraging in places with more plentiful fruits.

We therefore tested three hypotheses regarding the effects of decreasing human activity on the foraging behaviors of frugivorous omnivores in urban forests based on the previous study (Osugi et al., 2019): (1) diurnal foraging behavior would increase in 2020 compared to 2019 due to less human activity, (2) the duration of foraging per visit to fruiting trees would not change between day and night but it would be longer in 2020 than in 2019 due to less human activity, with these hypotheses are based on previous comparisons between urban forest and mountainous forest; and (3) foraging in 2020 would be more frequent at more trees with higher fruiting abundance than in 2019, as individuals would prioritize foraging at trees with a higher fruit production when there was less risk from human disturbance. This hypothesis is based on the assumption that the amount of fruit production will become a priority factor for selecting fruiting trees in the same way that many animals select fruiting trees as human activity declines, even in urban forests (e.g., Davidar & Morton, 1986).

## 2 METHODS

### 2.1 Study area

We conducted our study at a university campus (International Christian University; hereafter ICU) in Mitaka, Tokyo, Japan (located at 35°41′ N, 139°28′ E), covering about 62 ha at an elevation of 60 m a.s.l. While a 15‐ha nature‐protected area to preserve biodiversity is located on the campus, this study was conducted outside the protected area of the campus. Five terrestrial mammal species inhabit the study area (Kamito et al., 2019): domestic cat (*Felis catus*), Japanese badger, masked palm civet (*Paguma larvata*), northern raccoon (*Procyon lotor*), and raccoon dog.

### 2.2 Target tree and mammal species characteristics

Ginkgo (*Ginkgo biloba* L.) is a coniferous tree that reaches a height of 20–30 m and bears orange‐colored, fleshy fruit with a pungent odor, that ripens in autumn (fruit diameter; 10–20 mm) (Koike & Masaki, 2019). Muku (*Aphananthe aspera* Thunb.) is a broadleaf deciduous tree that grows to approximately 15–20 m in height and bears dark purple fruit that ripens in autumn (fruit diameter; 7–12 mm) (Koike & Masaki, 2019).

In 2020, we selected 10 ginkgo and nine muku trees with the same attributes as study trees in 2019 (Osugi et al., 2022). All of the trees bore fruits, and were located on flat landscape with a diameter at breast height of 30–40 cm. However, since access to the interior of the campus was severely restricted in 2020, we chose trees located within the boundaries of the campus, thus there are different individual trees from Osugi et al. (2022).

The raccoon dog (weight = 4.1 kg ± 0.9 SD; body length = 74.5 cm ± 6.1 SD), which is primarily monogamous (Ohdachi et al., 2009), and the Japanese badger (weight = 7.7 km ± 1.3 SD; body length = 78.7 cm ± 4.9 SD), which forms family groups of mother and cubs as the social unit (Ohdachi et al., 2009), are widely distributed in forests from mountainous to urban forests (Mise et al., 2016; Soga & Koike, 2013). Raccoon dogs and Japanese badgers are opportunistic omnivores that feed mainly on fruits and invertebrates (Koike et al., 2008, 2012, 2023), but neither of these species is particularly adept at climbing trees so they primarily forage on fallen fruit. Previous studies show that they prefer to feed on fruit of muku and ginkgo trees in the fall in urban forests (e.g., Koike & Masaki, 2019), although Japanese badgers more eat more animal materials than fruits (e.g., earthworms; Koike et al., 2012).

### 2.3 Recording foraging behavior

We installed a video camera trap (Ltl Acorn Ltl‐6210MC, Zhuhai Ltl Acorn Electronics Co., Ltd., Zhuhai, China) under each tree in the study to capture the activity of animals on the forest floor (see Osugi et al., 2022 for details). From the videos, we recorded the mammal species feeding on fallen fruit, the number of foraging visits per species per day (frequency of foraging visits), and the duration of foraging per visit per animal (duration of foraging: total time foraging + feeding only at each visit).

The cameras ran from September 21 to December 19, 2020, for a combined total of 900 days for the ginkgo trees and a combined total of 810 days for Muku trees. The actual study period was from when the first fruits fallen observed at each tree, to when the almost fruits fallen at each tree. Sunset in late September and late November is at 5:30 p.m. and 4:30 p.m. respectively, and sunrise in late September and late November is at 5:30 a.m. and 6:30 a.m. respectively.

### 2.4 Measuring the environmental characteristics of the study trees

We measured three environmental characteristics known to affect the selection of fruit trees by frugivorous animals for each ginkgo and muku tree in our study at ICU (see Osugi et al. [2022] for details).

First, the shortest distance from the study tree to the boundary of the protected area (DP) was measured by QGIS (v 3.4.10). This factor served as an indicator of the ease of moving between the study tree and a preferred resting habitat.

Second, the degree of shelter (DS, i.e., how easily the animals could hide near the study trees using vegetation or other structures) given by each tree. This factor was an index for how difficult it would be to detect foraging animals from the surrounding area. The shortest distance between the study trees and walkways was approximately 10 m based on the recorded footage of raccoon dogs and badgers foraging on the forest floor in the presence of passers‐by. Therefore, we defined the degree of shelter as the ratio of the 360‐degree field of view within a 10‐m radius from the study tree that was blocked by structures or vegetation. To measure this ratio, we placed surveying poles 10 m away from the study tree to the north, south, east, and west. The angle of view from the base of the tree that was blocked within each quarter‐circle area was measured by calculating the difference between the azimuth angles at both ends of ≥30‐cm structures and vegetation (based on the body heights of raccoon dogs and Japanese badgers). The degree of shelter at each study tree was then determined by converting the resulting ratio into a four‐level rating from 0 to 3, where 0 was 0 to less than 25% shelter, 1 was 25 to less than 50% shelter, 2 was 50 to less than 75% shelter, and 3 was 75 to 100% shelter.

Third, the quantity of fruit production (FP) of the study tree. We estimated the amount of fruit production of each study tree with binoculars, according to the method by Nakajima et al. (2015). We randomly looked over the tree canopy using binoculars and estimated fruit production based on the number of fruits counted within a fixed period (e.g., 30 s) and the area of the tree canopy, which was estimated from the diameter of a vertical two‐dimensional circle. We counted the fruit production on 25 September for ginkgo trees and 1 October for muku trees in 2020, when the color of the fruit exocarp began to change and they were easier to detect.

### 2.5 Statistical analyses

To test our three hypotheses, we conducted analyses as described below. We performed all analyses in Program R (ver. 4.0.3; R Core Team, 2022), and in each analysis we considered *p* < .05 to be statistically significant.

First, to evaluate the effect of decreasing human activity on the frequency and time of foraging behavior at fruit trees, we analyzed how activity patterns of foraging visits change by the year (2019 vs. 2020). To understand the activity patterns of our two study species, we calculated the frequency of foraging visits per day during each hour (0:00–24:00) and analyzed activity patterns through the *overlap* package (Meredith & Ridout, 2021). We computed the overlap coefficient of temporal activity patterns (Δ, which ranges from 0 [no overlap] to 1 [complete overlap]) for each animal–tree species pair (raccoon dogs–gingko, raccoon dogs–muku, Japanese badgers–gingko, and Japanese badgers–muku). We used Δ_1_ estimator, following the recommendation of Ridout and Linkie (2009) because the smaller of our samples had <75 records, and calculated confidence intervals from 10,000 smoothed bootstrap samples. We also calculated the frequency of foraging visits per day each hour for each animal–tree species pair and estimated associated 95% confidence intervals by empirical bootstrapping using the *activity* package (Rowcliffe, 2014). We conducted a Wald Test (Rowcliffe, 2014) with data of 95% confidence intervals to assess the difference in activity between years for each animal–tree species pair, and considered changes in activity to have occurred when Wald Test P‐values are below 0.05.

Second, to test if the duration of foraging changed with the lockdown and determine temporal factors driving the duration of foraging per visit, we used generalized linear mixed models (GLMMs). We created GLMMs with the *lme4* package of R (Bates et al., 2015) to perform analyses for each animal species. We set the duration of foraging (in seconds) as the response variable, we set hour, year, and the hour × year (interaction term) as fixed effects and study tree ID as a random effect. Because there may be uncertain factors associated with each study tree that influence mammals' selection to stay under fruiting trees, we set study tree ID that include the tree species (i.e., every study tree ID is named explicitly with each tree species) as a random effect. The model used Gamma log‐link for both species. We compared the models in an AIC framework (Burnham & Anderson, 2003) using AICc weight (*w*) after removing models that did not converge from our comparisons, and considered any model with <0.90 cumulative *w* to be a top model (Burnham & Anderson, 2003).

Third, we analyzed the factors that could explain trees the animals selected to forage from and how the effects of environmental characteristics affected tree selection in each year using a GLMM. We set the model for each year using the frequency of foraging visits by each species beneath the tree canopy at each tree as the response variable, and DP, DS, and FP as explanatory variables. We conducted GLMM with a Poisson link for raccoon dog and a binomial link Japanese badger due to the frequency of foraging visits of raccoon dog having a discrete data structure without overdispersion, and that of Japanese badger having a binary data structure. We used *lme4* package (Bates et al., 2015) for the models. We set study tree ID as a random effect is that there may be the factors influencing the selection of fruiting trees by mammals other than the environmental factors related to each study tree of this study. We analyzed each animal–tree species pair and compared the models in an AIC framework as described above.

## 3 RESULTS

### 3.1 Temporal activity

In 2019, prior to the lockdown, most foraging visits to ginkgo by raccoon dogs were nocturnal (98.9%), and all foraging visits by Japanese badgers were nocturnal (100%). All foraging visits to muku by both raccoon dogs and Japanese badgers were nocturnal (100%; Table 1). In contrast, during the lockdown in 2020, both species were observed foraging during the day to a significantly greater degree (with the exception of badgers foraging on ginkgo). Specifically, raccoon dogs exhibited diurnal foraging at 28.9% of visits to ginkgo (*p* < .001) and at 18.4% of visits to muku (*p* < .001), while Japanese badgers exhibited diurnal foraging at 16.7% of visits to ginkgo (*p* = .255) and at 7.8% of visits to muku (*p* < .001; Table 1).

**TABLE 1:** Number of animals visiting to forage gingko and muku trees (mean ± SD) in 2019 and 2020. The data of 2019 were from Osugi et al. (2022).

	Ginkgo trees		Muku trees	
	Raccoon dogs	Japanese badgers	Raccoon dogs	Japanese badgers
(a) 2019				
Visits over the entire survey period				
Total	316	12	81	132
Nighttime	313	12	81	132
Day time	3	0	0	0
Visits per day (animals/day)				
Total	0.54 ± 0.74	0.02 ± 0.04	0.14 ± 0.11	0.23 ± 0.31
Night time (proportion)	0.53 ± 0.73 (98.9%)	0.02 ± 0.04 (100%)	0.14 ± 0.11 (100%)	0.23 ± 0.31 (100%)
Day time (proportion)	0.01 ± 0.02 (1.1%)	0 (0%)	0 (0%)	0 (0%)
(b) 2020				
Visits over the entire survey period				
Total	324	18	87	155
Nighttime	228	15	71	140
Day time	96	3	16	15
Visits per day (animals/day)				
Total	0.45 ± 0.28	0.03 ± 0.02	0.12 ± 0.04	0.27 ± 0.02
Night time (proportion)	0.32 ± 0.21 (71.1%)	0.02 ± 0.02 (83.3%)	0.10 ± 0.04 (81.6%)	0.25 ± 0.06 (92.2%)
Day time (proportion)	0.14 ± 0.07 (28.9%)	0.00 ± 0.01 (16.7%)	0.02 ± 0.04 (18.4%)	0.02 ± 0.01 (7.8%)

*Note*: The proportions (%) represent the proportion of animals visiting the experimental trees to forage at night (5:00 p.m.–6:00 a.m.) and during the day (6:00 a.m.–5:00 p.m.).

**FIGURE 1:**
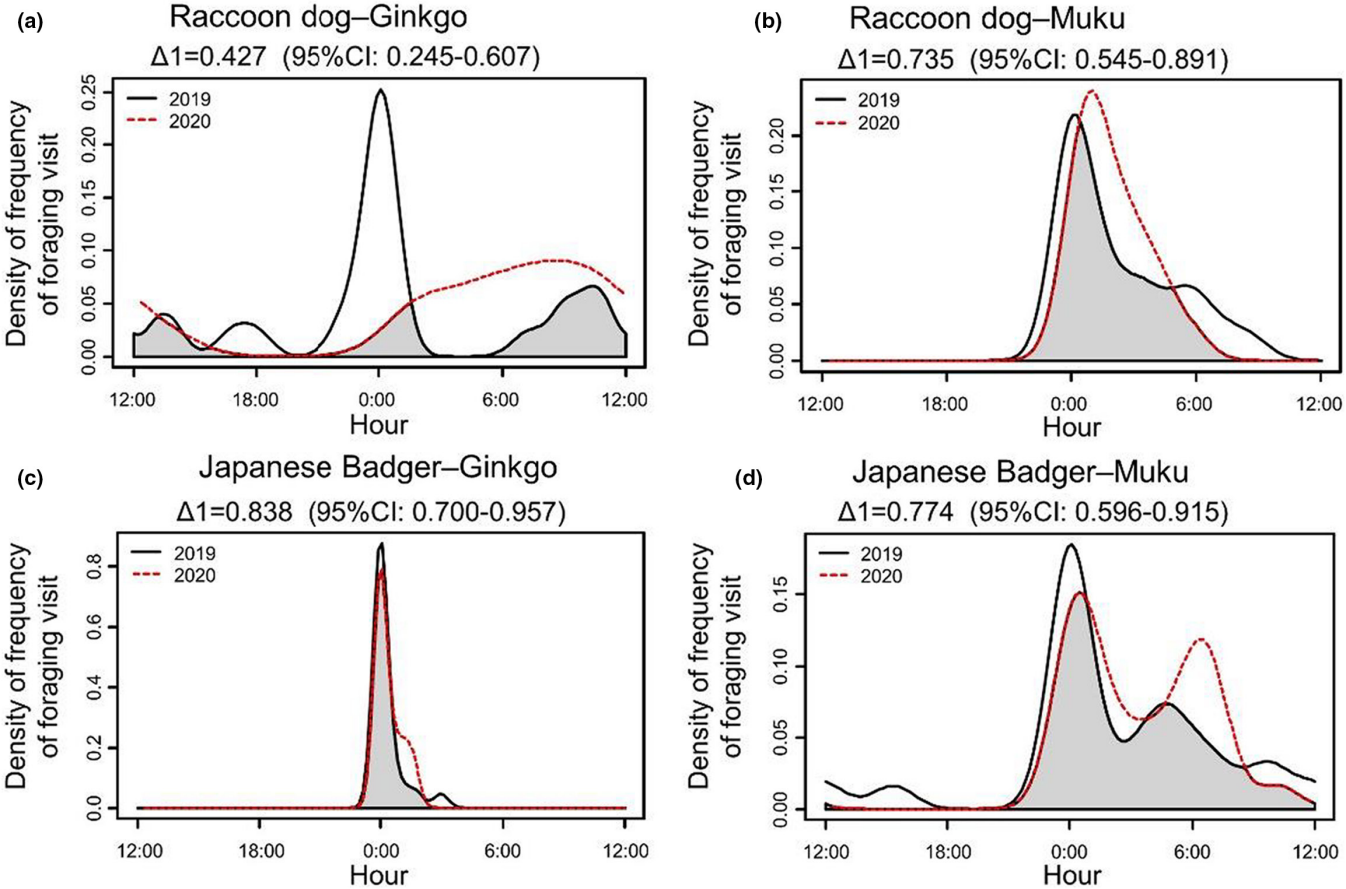
Interactive effect of hour and year (black line is 2019 and red dotted line is 2020) on number of animals that visited trees for foraging. The upper figures are raccoon dogs (a: Ginkgo trees, b: Muku tree) and lower figures are Japanese badger (c: Ginkgo trees, d: Muku trees). Gray shared areas indicate the coefficient of overlaps (Δ_1_) of the two density estimates.

Based on our temporal overlap analyses, the most substantial change in activity patterns between 2019 and 2020 was by raccoon dogs at ginkgo trees (Δ_1_ = 0.427; Wald test w = 10.792, *p* = .001; Figure 1a). Raccoon dogs at muku also showed variation but there is no significant difference between years (Δ_1_ = 0.735; Wald test w = 0.059, *p* = .807; Figure 1b). Japanese badgers foraging at both ginkgo and muku showed less variation in activity patterns between 2019 and 2020 (ginkgo: Δ_1_ = 0.838; Wald test w = 0.073, *p* = .787; muku: Δ_1_ = 0.774; Wald test w = 0.220, *p* = .639; Figure 1c,d).

### 3.2 Duration of foraging

For the temporal factors affecting the duration of foraging of raccoon dog, our top models were year (*w* = 0.57) and year + hour (*w* = 0.25; Table 2, Table S1). In both models, the effect of year was significantly positive (model 1 *ß* = .297, *p* = .003; model 2 *ß* = .298, *p* = .002; Table S1). In the second model, the effect of hour was positive but not statistically significant (*ß* = .002, *p* = .553; Table S1).

**TABLE 2:** Model selection results of top a‐priori models ranked by their AICc weight (*w*) for temporal factors on duration of foraging visit by raccoon dog and Japanese badger on ginkgo and muku trees.

Rank	Variables	*df*	AICc	∆AICc	*w*	Cumulative *w*
Raccoon dog						
1	Year	4	8675	0	0.57	0.57
2	Year + hour	5	8676.7	1.67	0.25	0.81
Japanese badger						
1	Null	3	2885.5	0	0.42	0.41
2	Year	4	2886.4	0.85	0.27	0.68
3	Hour	4	2887.6	2.05	0.15	0.83

For the temporal factors affecting the duration of foraging of Japanese badger, our top models were null (*w* = 0.41), year (*w* = 0.27) and hour (*w* = 0.15; Table 2, Table S1). In the second model, the effect of year was positive but not statistically significant (*ß* = .200, *p* = .286; Table S1). In the third model, the effect of hour was negative but not statistically significant (*ß* = −.0001, *p* = .986; Table S1).

### 3.3 Frequency of foraging visits

In 2020, the environmental factors affecting frequency of foraging visits by raccoon dog on ginkgo, our top models were FR (*w* = 0.41), FR + DP (*w* = 0.21), and the full model (*w* = 0.16; Table 3, Table S2). In the first model, the effect of FR had a significant positive effect (*ß* = .699, *p* < .001; Table S2). In the second model, the effect of FR had a significant positive effect (*ß* = .675, *p* < .001; Table S2), but the effect of DP was negative but not statistically significant (*ß* = −.173, *p* = .378; Table S2). In the third model, the effect of FR had a significant positive effect (*ß* = .674, *p* < .001; Table S2), but the effects of DP and DS were negative but not statistically significant (*ß*_DP_ = −.344, *p*_*DP*_ = 0.124; *ß*_DS_ = −.285, *p*_*DS*_ = 0.202; Table S2). For the raccoon dog–muku pair, our top models were FR (*w* = 0.28), FR + DP (*w* = 0.18), null (*w* = 0.14), FR + DS (*w* = 0.11), DP (*w* = 0.10), and full model (*w* = 0.07; Table 3, Table S2). In the first model, the effect of FR had a significant positive effect (*ß* = .239, *p* = .043; Table S2). In the second model, the effect of FR had a positive but weakly significant effect (*ß* = .213 *p* = .056; Table S2). The effect of DP was negative but not statistically significant (*ß* = −.133, *p* = .281; Table S2).

**TABLE 3:** Model selection results of top a‐priori models ranked by their AICc weight (*w*) for environmental factors on frequency of foraging visits by raccoon dog and Japanese badger on ginkgo and muku trees in 2020.

Rank	Variables	*df*	AICc	∆AICc	*w*	Cumulative *w*
Raccoon dog–ginkgo						
1	FR	3	1189.5	0	0.41	0.41
2	FR + DP	4	1190.8	1.29	0.21	0.62
3	FR + DP + DS	5	1191.3	1.81	0.16	0.78
Raccoon dog–muku						
1	FR	3	596.5	0	0.28	0.28
2	FR + DP	4	597.4	0.92	0.18	0.46
3	Null	2	597.8	1.35	0.14	0.60
4	FR + DS	4	598.4	1.91	0.11	0.71
5	DP	3	598.5	1.99	0.10	0.81
6	FR + DP + DS	5	599.3	2.79	0.07	0.88
Japanese badger–ginkgo						
1	FR	3	163.1	0	0.35	0.35
2	FR + DP	4	163.6	0.46	0.28	0.63
3	FR + DP + DS	5	165	1.95	0.13	0.76
4	FR + DS	4	165.1	1.96	0.13	0.89
Japanese badger–muku						
1	Null	2	692.7	0	0.36	0.36
2	DS	3	694.1	1.4	0.18	0.54
3	DP	3	694.7	2.02	0.13	0.67
4	FR	3	694.7	2.02	0.13	0.80
5	FR + DS	4	696	3.34	0.07	0.86

Abbreviations: DP, distance from protected area; DS, degree of shelter; FP, fruit production.

For the environmental factors affecting frequency of foraging visits by Japanese badger on ginkgo, our top models were FP (*w* = 0.35), FP + DP (*w* = 0.28), the full model (*w* = 0.13), and FP + DS (*w* = 0.13; Table 3, Table S2). In the first model, the effect of FP had a significant positive effect (*ß* = .599, *p* = .006; Table S2). In the second model, the effect of FP had a significant positive effect (*ß* = .522, *p* = .017; Table S2), but the effect of DP was negative but not statistically significant (*ß* = −.351, *p* = .235; Table S2). In the third model, the effect of FP had a significant positive effect (*ß* = .606, *p* = .020) and the effects of DP and DS were also positive but non‐significant (*ß*_DP_ = −.511, *p*_*DP*_ = 0.157; *ß*_DS_ = −.243, *p*_*DS*_ = 0.465; Table S2). For the Japanese badger–muku pair, our top models were null (*w* = 0.36), DS (*w* = 0.18), DP (*w* = 0.13), FR (*w* = 0.13), and FR + DS (*w* = 0.07; Table 3, Table S2). In the second model, DS was negative but not statistically significant (*ß* = −.362, *p* = .425; Table S2). In the third model, the effect of DP was negative but not statistically significant (*ß* = −.022, *p* = .963; Table S2).

## 4 DISCUSSION

Previous studies have predicted that the decline in human activity associated with the COVID‐19 will result in changes in animal behavior across activities and typologies such as (i) activity schedules, (ii) density, (iii) exploratory behaviors, (iv) movement dynamics, (v) ranging and resource use, (vi) vocalizations, and (vii) vigilance. And, it has been pointed out that spatial and temporal comparisons of these typologies are necessary to assess the effects of declines in human activity (Montgomery et al., 2021). Since the pandemic, changes in animal responses have been examined in studies for changes in wildlife–vehicle collisions, observations by camera traps, and the records of citizen science data (Jasińska et al., 2022; Łopucki et al., 2021; Manenti et al., 2020). However, there have been no detailed studies of changes in the behavior of urban mammals. In this study, by comparing behaviors before (2019) and after the decline in human activity (2020), we were able to show specific behavioral changes in the foraging behavior of urban mammals, especially raccoon dogs, due to changes in human activity after the voluntary lockdown due to the COVID‐19 pandemic.

We hypothesized that raccoon dogs and Japanese badgers would shift their temporal activity patterns to be more diurnal when released from competitive pressures of humans due to COVID‐19 restrictions. Mesocarnivores (small‐ to mid‐sized carnivore species) are known to shift their temporal patterns depending on a variety of factors and are often completely nocturnal in urban areas (avoidance of dominant carnivores: Frey et al., 2022; Suraci et al., 2021; Wang et al., 2015; avoidance of humans: Gaynor et al., 2018; Riley et al., 2003; Tigas et al., 2002; prey activity: Leighton et al., 2021). We found that raccoon dogs became more diurnal during their foraging behavior with substantial shifts in their activity patterns in 2020, while Japanese badgers exhibited diurnal foraging behavior in 2020 after never exhibiting it in 2019. Although the diurnal rhythms of both species are essentially nocturnal (Ohdachi et al., 2015), raccoon dogs foraging ginkgo fruit in particular are known to forage even during the day in mountainous forests where human activity is low (Osugi et al., 2019). In other countries with required lockdowns, increases in wildlife sightings, especially in urban areas, was thought to be due to animals changing their time of activity in response to the decrease in human activity caused by the Covid‐19 (e.g., Silva‐Rodríguez et al. 2021). In the previous study, Japanese badgers were also more active at night, even in mountainous areas (Osugi et al., 2019), so it was probably unlikely for them to significantly change their activity pattern even when human activity decreased. Based on the above, our hypothesis 1 was partially supported and mostly supported in raccoon dogs.

Duration of foraging time per visit was longer in 2020 than in 2019 for raccoon dogs and Japanese badgers, supporting our hypothesis 2. It is known that human activities and disturbance (such as light at night) curtail the foraging times of some felids species (Kerley et al., 2002; Smith et al., 2017), and can also change the ways in which species forage and how readily prey can avoid predators (Biebouw & Blumstein, 2003; Buchanan, 1993; Dwyer et al., 2013; Wakefield et al., 2015). In general, both of our study species are known to spend significantly shorter durations foraging per visit in heavily populated urban areas than in mountainous areas where there is no human presence (Osugi et al., 2022). This is because foraging behavior in these risky areas may be perceived as dangerous for animals, so animals keep foraging visits as short as possible. The increase in duration of foraging per visit in 2020 is likely a result of reduced human activity, and possibly because less vigilance was needed due to the decrease in human activity and led to a longer foraging time per meal for the major food items (fallen fruits). On the other hand, if these two mammal species have consumed anthropogenic food (e.g., garbage), the decrease in human activity could result in a decrease in anthropogenic food, thereby increasing foraging time for natural food items (Stofberg et al., 2019). However, this possibility would be unlikely given that these two mammal species do not generally eat anthropogenic food in this area (Osugi unpublished data). And, if we assume that the density of the population did not change between the two years, the decrease in human activity may have resulted in an increase in the amount of food consumed per visit. This may have allowed both species to eat at fewer foraging sites, thus allowing for more efficient foraging activities. If this is the case, the changes in foraging activity associated with reduced human activity may also affect the reproductive and parental success of both species the following spring.

Perhaps the most profound effect of the reduction in human activity were the factors that both species selected for in trees when foraging. In 2019, both species chose trees that provided greater cover rather than trees with high fruiting volume (Osugi et al., 2022). However, in 2020, when human activity declined, greater cover was not the most important factor (except when feeding on muku fruits by Japanese badger) for both species when selecting trees to forage from. This result suggests that the decline in human activity and that the abundance of food resources became a higher priority than the ability to hide from humans, similar to the selection of fruiting trees by frugivore animals in general, thus, Hypothesis 3 was supported.

## AUTHOR CONTRIBUTIONS

**Shigeru Osugi:** Investigation (lead). **Seungyun Baek:** Methodology (supporting). **Tomoko Naganuma:** Investigation (supporting). **Kahoko Tochigi:** Formal analysis (lead). **Maximilian Allen:** Writing – review and editing (supporting).

## CONFLICT OF INTEREST

The authors declare no competing interests.

## Supporting information


Table S1 and S2

## Data Availability

Data available on request from the authors
